# Automated detection of intracranial aneurysms using skeleton-based 3D patches, semantic segmentation, and auxiliary classification for overcoming data imbalance in brain TOF-MRA

**DOI:** 10.1038/s41598-023-38586-9

**Published:** 2023-07-25

**Authors:** Sungwon Ham, Jiyeon Seo, Jihye Yun, Yun Jung Bae, Tackeun Kim, Leonard Sunwoo, Sooyoung Yoo, Seung Chai Jung, Jeong-Whun Kim, Namkug Kim

**Affiliations:** 1 Healthcare Readiness Institute for Unified Korea, Korea University Ansan Hospital, Korea University College of Medicine, 123 Jeokgeum-ro, Danwon-gu, Ansan City, Gyeonggi-do 15355 Republic of Korea; 2 Department of Convergence Medicine, University of Ulsan College of Medicine, Asan Medical Center, Seoul, Republic of Korea; 3 Department of Radiology, University of Ulsan College of Medicine & Asan Medical Center, Seoul, Republic of Korea; 4 Present Address: Department of Radiology, Seoul National University College of Medicine, Seoul National University Bundang Hospital, Seongnam, Republic of Korea; 5 Department of Neurosurgery, Seoul National University Bundang Hospital, Seongnam, Republic of Korea; 6 Department of Radiology, Seoul National University Bundang Hospital, Seongnam, Republic of Korea; 7 Healthcare ICT Research Center, Office of eHealth Research and Businesses, Seoul National University Bundang Hospital, Seongnam, Republic of Korea; 8 Department of Otorhinolaryngology, Seoul National University College of Medicine, Seoul National University Bundang Hospital, Seongnam, Republic of Korea

**Keywords:** Biomedical engineering, Computational biology and bioinformatics, Predictive markers, Neurological disorders

## Abstract

Accurate and reliable detection of intracranial aneurysms is vital for subsequent treatment to prevent bleeding. However, the detection of intracranial aneurysms can be time-consuming and even challenging, and there is great variability among experts, especially in the case of small aneurysms. This study aimed to detect intracranial aneurysms accurately using a convolutional neural network (CNN) with 3D time-of-flight magnetic resonance angiography (TOF-MRA). A total of 154 3D TOF-MRA datasets with intracranial aneurysms were acquired, and the gold standards were manually drawn by neuroradiologists. We also obtained 113 subjects from a public dataset for external validation. These angiograms were pre-processed by using skull-stripping, signal intensity normalization, and N4 bias correction. The 3D patches along the vessel skeleton from MRA were extracted. Values of the ratio between the aneurysmal and the normal patches ranged from 1:1 to 1:5. The semantic segmentation on intracranial aneurysms was trained using a 3D U-Net with an auxiliary classifier to overcome the imbalance in patches. The proposed method achieved an accuracy of 0.910 in internal validation and external validation accuracy of 0.883 with a 2:1 ratio of normal to aneurysmal patches. This multi-task learning method showed that the aneurysm segmentation performance was sufficient to be helpful in an actual clinical setting.

## Introduction

Cerebral aneurysms are bulges in cerebral blood vessels that can leak or rupture, causing subarachnoid hemorrhage (SAH). An unruptured intracranial aneurysm is an abnormal focal expansion of an artery in the brain caused by the weakening of the vascular wall. Approximately 3% of healthy adults have an intracranial aneurysm^1^. Aneurysms account for 85% of all SAHs, with an average mortality rate of 51%, and one-third of survivors have long-term disabilities^2^. Rupturing of an intracranial aneurysm is a serious event with high mortality and morbidity^3^. There is active research into the identification of factors contributing to the risk of intracranial aneurysms developing, growing, and rupturing^4–6^.

Genetic factors, family history, female sex, and age are associated with an increased risk of aneurysm development. Additionally, the site, size, and shape of the intracranial aneurysm are more closely related to the risk of rupture^2,7,8^. Automated detection of intracranial aneurysms before symptoms occur can prevent death or other complications through intravascular or surgical treatment; however, detection of small aneurysms has proved difficult. Owing to the recent rapid development of deep-learning-based models, the ability to automatically detect and segment intraarterial aneurysms would be especially helpful in reducing the fatigue and increased workloads of radiologists.

Studies have proposed several approaches with conventional image processing techniques for the semi-automatic detection of aneurysms^9–12^. Convolutional neural networks (CNN) have demonstrated excellent performance in various visual tasks, including medical image analysis^13,14^. Several medical imaging tasks, including detection and segmentation, have been revolutionized by the application of deep learning algorithms, and they have shown dramatic improvements in multiple computer vision tasks. Additionally, several studies have investigated the automated detection of brain aneurysms with deep learning algorithms. Ueda et al. used a 2D patch-based ResNet architecture to detect aneurysms with time-of-flight magnetic resonance angiography (TOF-MRA)^15^. Stember et al. proposed a method to build a model that would predict the size of aneurysms after training the U-net architecture with 250 maximum intensity projections (MIPs) of MRA^16^. Nakao et al. developed a computer-assisted detection (CAD) system for intracranial aneurysms in MIPs of TOF-MRA images based on a CNN classifier^17^. Most methods have been developed using 2D or 2D-projection-like MIPs, which could be a limitation for detecting 3D aneurysms using 3D MRA.

Therefore, our study aims to develop and validate a CNN model with 3D patches for automatic detection and segmentation of intracranial aneurysms using TOF-MRA and a multi-center cohort. In addition, a preliminary assessment of the accuracy and clinical usefulness of the model was conducted.

## Materials and methods

### Dataset

This retrospective study was conducted in accordance with the Declaration of Helsinki and current scientific guidelines. The study protocol was approved by the Institutional Review Board Committee of Seoul National University Bundang Hospital (SNUBH), Seoul, Korea, which also waived the requirement for informed patient consent. Candidate patients were identified by searching the picture archive and communication system from May 2011 to December 2017. A contrast agent was used to obtain all angiographic data. The final dataset included studies collected using two different modalities: a Siemens Axiom Artis (Siemens Healthcare, Erlangen, Germany), and a GE Innova IGS 630 (GE Healthcare, Chicago, US). The acquisition parameters were as follows: rage of slice thickness, 0.2–0.5 mm, matrix size, 1024 × 1024 and range of voxel size, 0.2–0.7 ${\mathrm{mm}}^{3}$.

A total of 154 3D TOF-MRA datasets with intracranial aneurysms were acquired from SNUBH. Out of a total of 154 patients, we divided into 120 for training, 19 for validation, and 15 for testing. The patient’s ages ranged from 32 to 76 years (with a mean age 53.90 $\pm$ 12.97 years.) The gender distribution was 70% female and 30% male. According to the Korean Classification of Diseases (KCD) diagnostic codes, the majority of patients (150 individuals) were diagnosed with unruptured cerebral aneurysms. There was also one case of non-aneurysmal anatomical variant and three cases of subarachnoid hemorrhage due to posterior communicating artery aneurysms. The size of the aneurysms ranged from 1.8–32.6 mm, with a mean size of 2.6 mm ± 1.9. The distribution included 130 patients with very small aneurysms of less than 5 mm, 21 patients with small aneurysms ranging from 5 to 10 mm, and 3 patients with large aneurysms greater than 10 mm in size. The ground-truth masks of the intracranial aneurysm regions were delineated by a neuroradiologist (> 10 years of experience) who semi-automatically defined masks on the 3D contrast-enhanced T1 weighted images using manual segmentation with a segmentation threshold using MITK software (MITK, www.mitk.org, German Cancer Research Center)^18^. All segmented images were validated by a neuroradiologist (> 18 years of experience). It took 15–20 min to make the reference mask for each patient. To generate the training dataset, both negative (no aneurysm) and positive (with aneurysm) patches were extracted from the vessel skeleton of TOF-MRA volume. Specifically, positive patches for each aneurysm were randomly extracted in a non-centered fashion around the aneurysm center, always ensuring that the manual mask was completely included in the patch. The patch size was 64 × 64 × 64, and values of the ratio of patches with and without aneurysms ranged from 1:1 to 1:5 for evaluating ablation studies on imbalances. Our patch size was determined experimentally, the results of which can be found in Supplementary Table S1. We have sourced a robust collection of 113 external datasets from TOF-MRA, as part of the Aneurysm Detection And segMentation (ADAM) challenge. This ADAM datasets consisted of individuals aged 24–75 years, with a median age of 55. Notably, females represented a substantial 75% of these subjects. The patient cohort for this challenge was thoughtfully curated from a larger pool at the University Medical Center (UMC) in Utrecht. The MRI scans in this study were conducted at UMC Utrecht in the Netherlands using a range of Philips scanners with field strengths of 1, 1.5 or 3 T. The TOF-MRAs provided variable voxel spacings in the image plane, between 0.195 to 1.04 mm, and a slice thickness that ranged from 0.4 to 0.7 mm. It should be noted that the acquisition protocol was not standardized, reflecting the clinical nature of the data, which was collected from various studies spanning the period from 2001 to 2019. The data included subjects with at least one untreated, unruptured intracranial aneurysm (UIA), as well as those without any intracranial aneurysm. The sample also contained subsets of individuals who were screened for UIAs due to a familial history of aneurysmal subarachnoid hemorrhage (aSAH). An interventional neuro-radiologist (> 10 years of experience) created the protocol for aneurysm annotation. The outline of every aneurysm was contoured on each axial slice of the TOF-MRA image. The annotated lines were always drawn from the neck level to the aneurysm's dome. All voxels whose volume was greater than 50% of the manually drawn contour's volume were included when the contours were transformed into binary masks. Untreated, undisrupted aneurysms were labelled 1, whereas the background was labelled 0.

### Pre-processing

**Figure 1:**
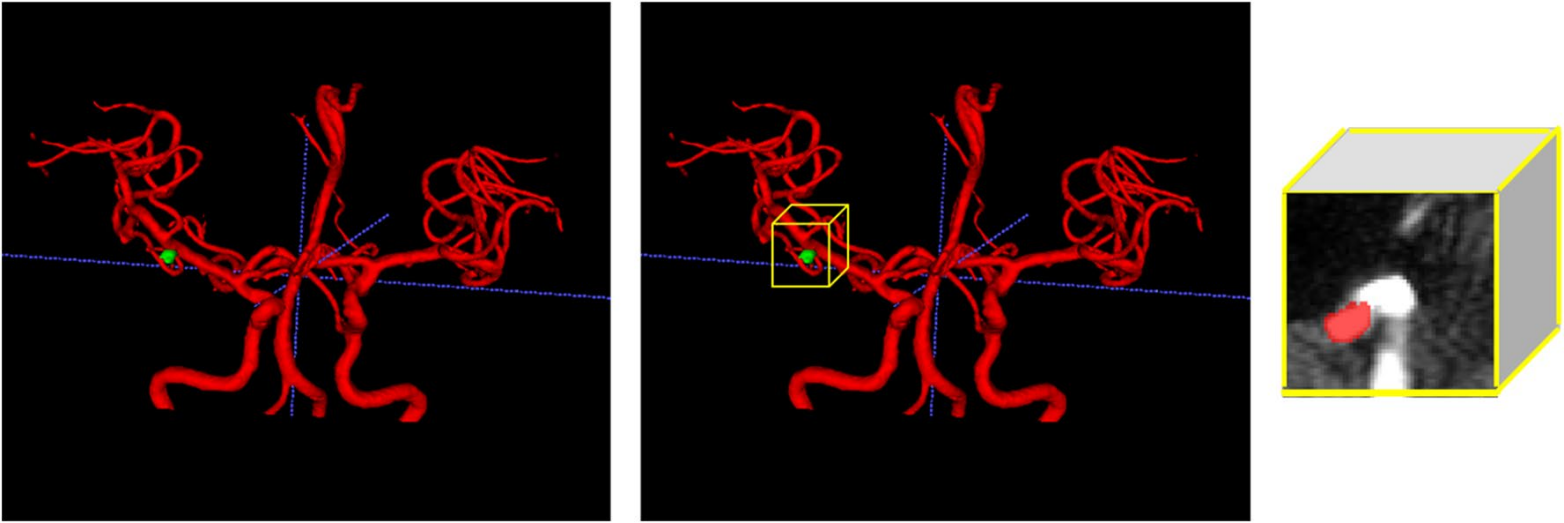
Vessel segmentation in the brain reduces dimensionality by pre-processing.

To enhance the learning capabilities of the deep learning model, pre-processing of brain MRA images is necessary. Additionally, in order to minimize the domain gap between the internal and external datasets, we performed the following preprocessing steps. First, signal intensity normalization was performed. The signal intensity of all MRA datasets was clipped to the [0.5, 99.5] percentiles of these intensity values, and then the mean and standard deviation of each dataset's intensity values were used to standardize the z-score for normalization. Second, to correct any intensity inhomogeneities caused by variations in scanner characteristics, patient positioning, N4 bias correction was applied. Last, the study of MR brain images requires preliminary processing to isolate the brain from extracranial or non-brain tissue, commonly referred to as skull stripping. This provides better detection or segmentation and direct visualization^19,20^. We removed the skull using a trained deep-learning bet model. Voxel spacing was resampled from an average value of 0.3 × 0.3 × 0.3 mm isotropic voxel size, and 3D patches sized 64 × 64 × 64 were extracted along the vessel skeleton in MRA^21^, as shown in Fig. 1. The vessel skeletonization proceeded as follows. A thresholding was applied to the original image and converted to binary format to isolate the region of interest and exclude the background, connected component labeling was used to identify and group large structures within the image, approximately 3000 pixels in size, and a region growth algorithm was run to iteratively add adjacent pixels to identify all pixels that comprise the same structure. To ensure structural continuity, we used a morphological closure operation to fill existing holes within the image. We concluded the procedure by iteratively applying the algorithm to each pixel and its 26 neighbors until no more pixels could be removed, completing the 3D skeletal representation of the image. To overcome the extreme imbalance between normal and aneurysm patches, these patches were augmented with horizontal and vertical flips, zooming, Rician and Gaussian noise, rotation, blurring, contrast, and gamma correction.

### Development of deep learning model

#### Network architecture

Several approaches were adopted to improve model performance. First, the proposed model was built based on the U-Net^22^, a well-proven network structure that has been used widely in medical image segmentation. Second, we employed a basic block structure rather than the stacked convolution layer; as a result, the residual connection improved the performance of the deep conventional network. Finally, the dual attention block was employed to force the network to focus on the informative region and features. We also compared the performance of the model to that of the most frequently employed 3D U-Net using the same training and testing data (Internal cohort 1), and our model had a significantly better performance, as shown in Table 1. 3D U-Net is a popular architecture for semantic segmentation^23^. It consists of a contracting and an expansive path. The contracting path adheres to the standard convolutional network architecture.

**Table 1:** Evaluation results of the internal dataset.

Model		Acc	Specificity	PPV	NPV	Sensitivity	DSC
3D U-Net		0.785**	0.668**	0.695**	0.798**	0.882*	0.277 ± 0.126
3D U-Net with auxiliary loss	1:1	0.885*	0.745**	0.798**	0.834*	0.884*	0.584 ± 0.122
	1:2	0.910	0.893	0.896	0.909	0.926	0.701 ± 0.217
	1:3	0.898	0.878	0.879	0.880	0.887*	0.775 ± 0.060
	1:4	0.887	0.864*	0.870	0.879*	0.885*	0.780 ± 0.177
	1:5	0.887	0.869*	0.875*	0.880	0.885*	0.767 ± 0.152
nnU-Net	0.802**	0.782**	0.786**	0.794**	0.798**	0.678 ± 0.175	

For comparisons between groups, t-tests were performed based on a 1:2 ratio, with *p*-values less than 0.05 denoted by ** and *p*-values less than 0.5 denoted by *to indicate statistical significance. Results with *p*-values less than 0.05 are considered statistically significant. Accuracy (Acc); predictive value (PPV); negative predictive value (NPV); dice similarity coefficients (DSC); three-dimensional (3D).

Two 3 $\times$ 3 $\times$ 3 convolutions (unpadded convolutions) are applied twice, one after the other, each time being followed by a rectified linear unit (ReLU) and a 2 $\times$ 2 $\times$ 2 max pooling operation with stride 2 for down-sampling. We must double the number of feature channels for each down-sampling step. The feature map is up-sampled for each step in the expansive path, then two 3 $\times$ 3 $\times$ 3 convolutions, each followed by a ReLU, a 2 $\times$ 2 $\times$ 2 convolutions (“up-convolution”) to cut the number of feature channels in half, and a concatenation with the correspondingly cropped feature map from the contracting path. The loss of boundary pixels in every convolution necessitates cropping. Each 64-component feature vector is mapped to the desired number of classes using a 1 $\times$ 1 $\times$ 1 convolution at the final layer. In total, the network has 23 convolutional layers. The nnU-Net increases the general applicability of U-Net to perform automated configuration for arbitrary new datasets^24,25^.

The nnU-Net architecture closely follows the original U-Net architecture, including various techniques such as cascaded strategy, residual connection, attention mechanisms, squeeze and excitation, and dilated convolutions. For the cascaded strategy, the first U-Net operates on down-sampled images, and the second is trained to refine the segmentation maps created by the former at full resolution. There are two main differences between the original U-Net and nnU-Net: first, it uses up-sampled layers in the decoding branch rather than transpose convolutions; second, nnU-Net does not include batch normalization layers.

To compare the nnU-Net and original U-Net, our modified 3D U-Net with dense blocks and auxiliary loss for classification was proposed. The auxiliary loss function and stochastic gradient descent algorithms could solve the problem of vanishing features^26^. Our network contains three loss functions, including two auxiliary losses and the principal loss. Auxiliary functions were used to train sub-models by extracting essential features to add loss functions to the intermediate layers in the deep neural network^27^.

#### Implementation details

In our network, auxiliary classifiers are used to prevent gradient loss during training and to improve convergence and learning results. This auxiliary classifier adds a loss function with binary-cross-entropy loss to the nodes along the decoder network of U-Net. The output mask derives the learned result using dice loss. At the last inference time, the loss used for the classifier and that used for segmentation are averaged. Additionally, we tuned our hyperparameters as follows: the training batch size was set to 16, batch normalization was used, the parametric ReLU was used as the activation function, Dice similarity coefficient (DSC) and Tversky focal loss and cross-entropy loss were provided as the loss function, and the number of training epochs was set to 500. Additionally, backpropagation algorithms along with optimization algorithms used ADAM set to 0.001. From the full dataset, a training set was created by adjusting the ratio of the patches with aneurysms to those without aneurysms from 1:1 to 1:5. The validation set was used to evaluate model performance during training and at the end of each epoch for hyperparameter optimization, and the test set was used for evaluation of the trained model, but not in training or validation.

### Statistical analysis

To assess the performance of automatic segmentation of intracranial aneurysms, evaluation metrics such as accuracy, sensitivity, positive predictive value (PPV), negative predictive value (NPV), and DSC were used. DSC is defined in Eq. (1), where ${V}_{GT} \mathrm{and }{ V}_{CNN}$ are the volume of ground truth and model prediction results, respectively. Sensitivity (also known as recall) is defined in Eq. (2), where TP is true positives and FM is false negatives^28^. PPV and NPV are defined in Eqs. (3) and (4)^29^.

1 $${\text{DSC}}:\frac{{2\left| {V_{GT} \cap V_{CNN} } \right|}}{{\left| {V_{GT} } \right| + \left| {V_{CNN} } \right|}}$$

2 $${\text{Sensitivity}}:\frac{TP}{{TP + FN}}*100$$

3 $${\text{PPV}}:\frac{TP}{{TP + FP}}*100$$

4 $${\text{NPV}}:\frac{TN}{{TN + FN}}*100$$

## Results

**Figure 2:**
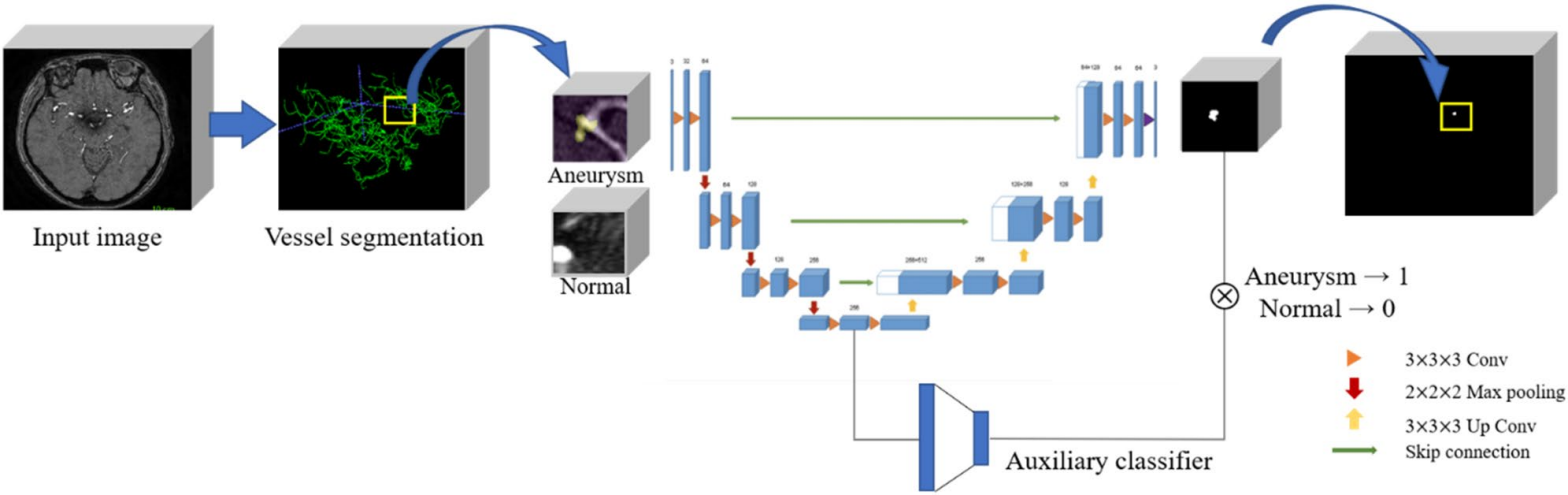
The proposed deep learning model (convolution; Conv).

For internal validation, 154 TOF-MRA images from SNUBH that used contrast agents were included. For external validation, 113 TOF-MRA images were included from the ADAM challenge datasets. The overall flowchart of our algorithm is shown in Fig. 2. The evaluation results for the detection and segmentation of intracranial aneurysms in internal datasets are shown in Table 1. The external validation results are described in Table 2. To accurately evaluate the model performance, we used accuracy, sensitivity, PPV, NPV, and DSC on both internal and external datasets. The highest accuracy was obtained when the ratio of normal to abnormal patches was 2:1 in internal datasets, as shown in Table 1. The accuracy, specificity, PPV, NPV, sensitivity, and DSC for the normal to abnormal ratio of 2:1 were 0.910, 0.893, 0.896, 0.909, 0.926, and 0.701 $\pm$ 0.217, respectively. After attaching an auxiliary classifier, the accuracy was improved relative to that without an auxiliary classifier. DSC increased as the normal to abnormal ratio increased from 1:1 and then decreased again at the 5:1 ratio^30,31^. In addition, to verify the robustness of the model against various aneurysm sizes, we calculated the mean accuracy for each aneurysm size in the external dataset as shown Table 3. Consistent with the model where the ratio of aneurysm to normal patch was 1:2, the highest accuracy of 0.885 was observed for sizes less than 5 mm.

**Table 2:** Evaluation results of the external datasets.

Model		Acc	Specificity	PPV	NPV	Sensitivity	DSC
3D U-Net		0.772	0.641	0.651	0.754	0.772	0.226 ± 0.301
3D U-Net with auxiliary loss	1:1	0.860	0.703	0.727	0.817	0.834	0.559 ± 0.209
	1:2	0.883	0.856	0.857	0.872	0.879	0.682 ± 0.114
	1:3	0.874	0.841	0.848	0.851	0.853	0.753 ± 0.106
	1:4	0.872	0.822	0.846	0.857	0.862	0.750 ± 0.194
	1:5	0.873	0.824	0.834	0.842	0.858	0.748 ± 0.173
nnU-Net	0.791	0.738	0.748	0.759	0.768	0.623 ± 0.198	

For comparisons between groups, t-tests were performed based on a 1:2 ratio, with *p*-values less than 0.05 denoted by ** and *p*-values less than 0.5 denoted by *to indicate statistical significance. Results with *p*-values less than 0.05 are considered statistically significant. Accuracy (Acc); predictive value (PPV); negative predictive value (NPV); dice similarity coefficients (DSC); three-dimensional (3D).

**Table 3:** The mean segmentation accuracy of the model by aneurysm size in the external validation set.

Aneurysm Size	3D U-Net	3D U-Net with auxiliary loss	nnU-Net				
			1:1	1:2	1:3	1:4	1:5
< 5 mm	0.778**	0.859*	0.885	0.875	0.870	0.871	0.798**
< 5–10 mm <	0.769**	0.859*	0.884	0.874	0.873*	0.875	0.787**
10 mm <	0.768**	0.861	0.881	0.874	0.873	0.873*	0.788**

For comparisons between groups, t-tests were performed based on a 1:2 ratio, with *p*-values less than 0.05 denoted by ** and *p*-values less than 0.5 denoted by *to indicate statistical significance. Results with *p*-values less than 0.05 are considered statistically significant.

**Figure 3:**
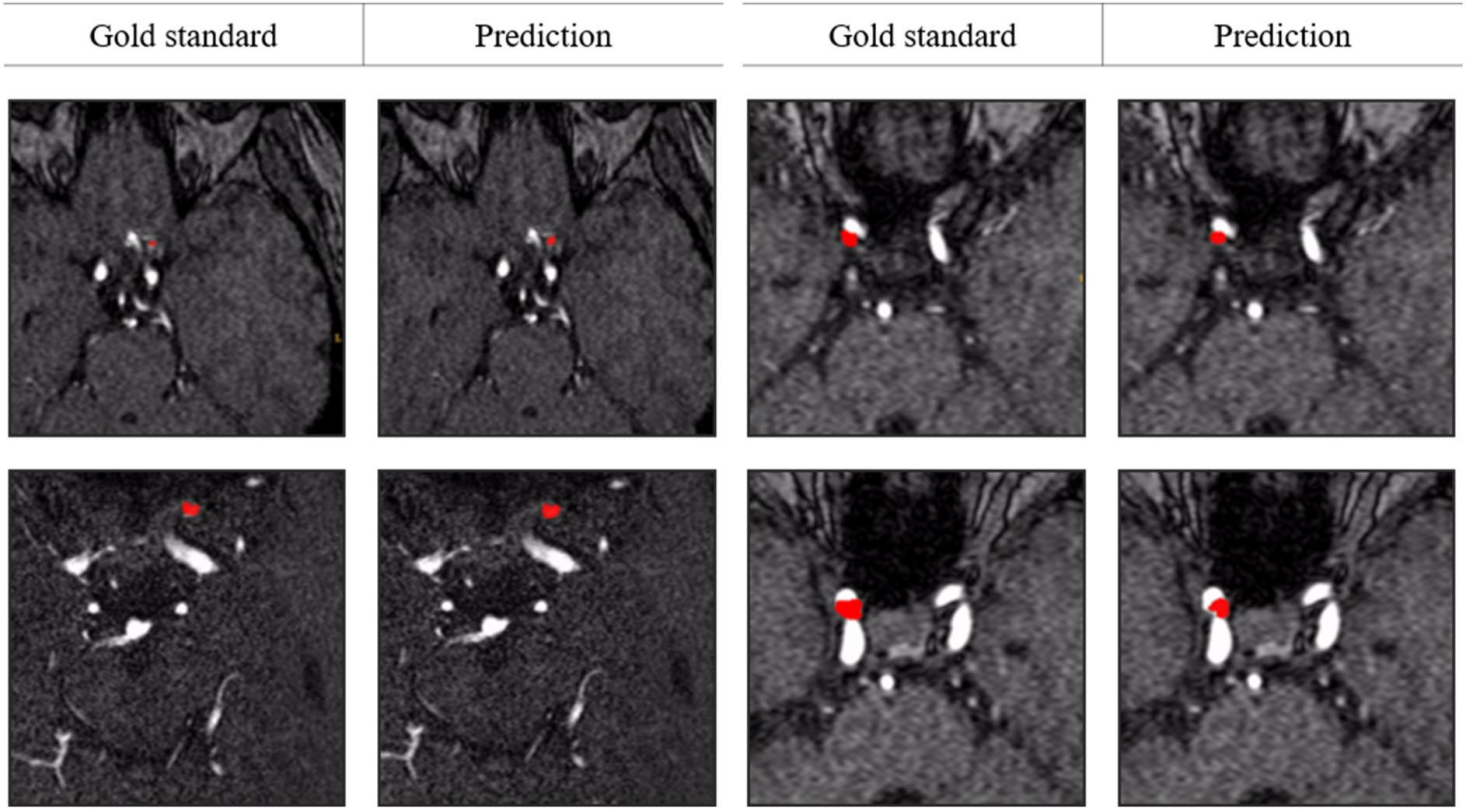
Example results of aneurysm segmentation in internal test datasets.

**Figure 4:**
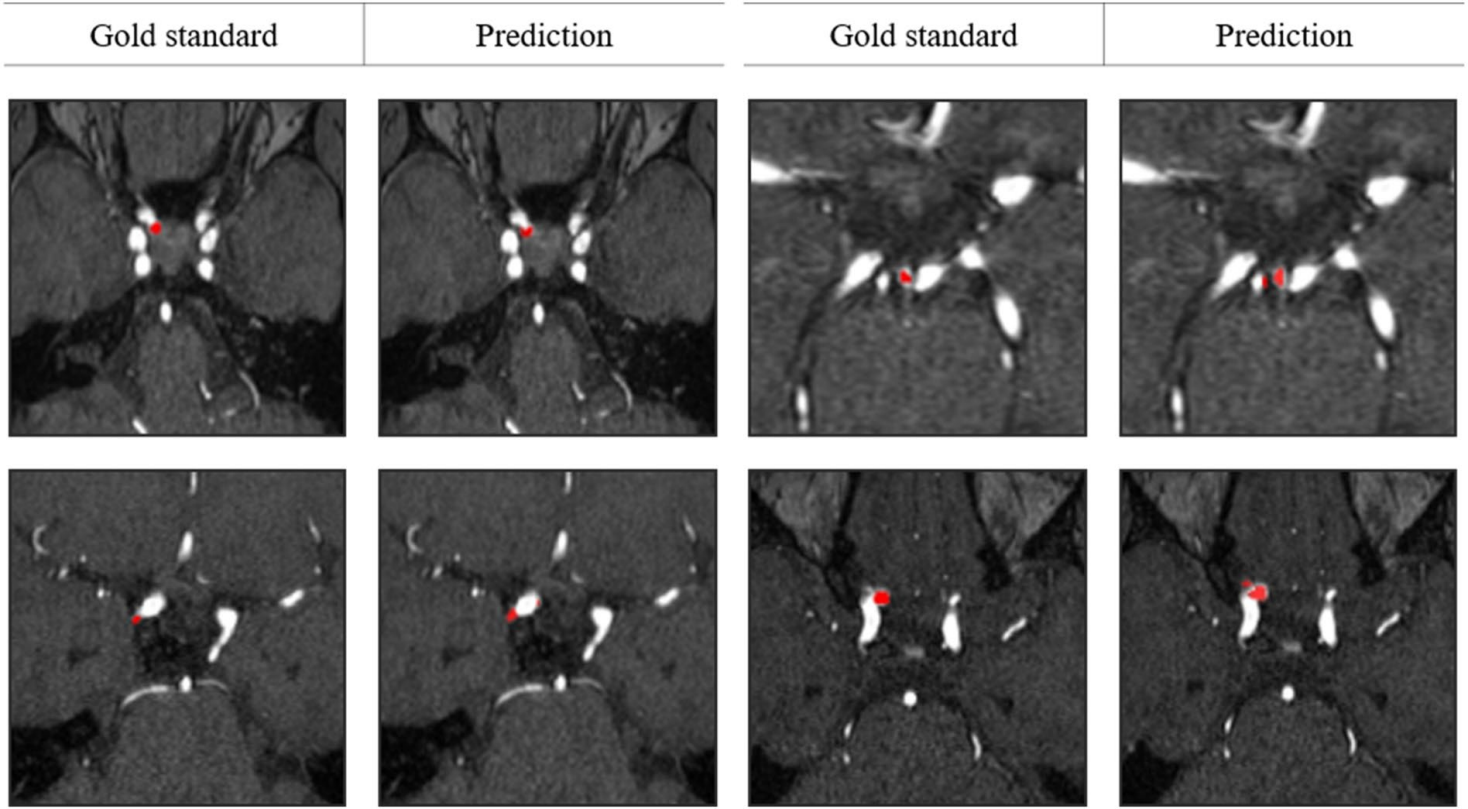
Example results of aneurysm segmentation in external datasets.

External validation results are shown in Table 2. Similar to the internal dataset results, model accuracy increased when the auxiliary loss was attached, and the highest accuracy was achieved when the ratio between normal and abnormal was 2:1. Examples of aneurysm detection are shown in Figs. 3 and 4 for internal and external validation datasets, respectively.

## Discussion

According to our study, CNNs have great potential for the reliable detection of intracranial aneurysms in 3D TOF-MRA. The model evaluated on a per-patient basis, with an overall mean DSC of our model was 0.755 ± 0.09, the sensitivity was 0.882, and the false positives (FP) was 0.305. The 3D patch-level technique demonstrated adequate aneurysm segmentation performance in terms of good sensitivity and minimal FP, which is applicable in the real-world clinical context. Segmentation of intracranial aneurysms, especially in smaller cases, is difficult, and misdiagnosis may result in poor clinical outcomes. Therefore, our deep learning model of automatic segmentation of intracranial aneurysms will be valuable to radiologists, who experience an increased workload and consequent fatigue. Fully automated CAD on intracranial aneurysms can help prevent diagnostic errors that are caused by fatigue or a lack of concentration in experts. We have demonstrated that our algorithm with multi-task learning can reliably detect aneurysms in non-invasive cranial imaging and requires only a limited number of training samples.

While previous studies only trained models on data with aneurysms^16^, this study applied training with mixed datasets that included individuals with and without aneurysms. Due to the severe data imbalance, the investigation was conducted while varying the ratio of aneurysmal to normal to patches from 1:1 to 1:5. Data ratios for stable learning that were saturated at 1:3 were also found. For aneurysms smaller than 10 mm^12^, the mean sensitivity of computed tomography angiography (CTA) was 70.4%; however, this study found an average sensitivity greater than 80%, even for small-sized aneurysms. Most aneurysms larger than 15 mm were segmented and detected^17^, but we were also able to detect and segment very small aneurysms (< 10 mm in size). Additionally, in order to assess the model's robustness in relation to various aneurysm sizes, we evaluated the mean accuracy for each aneurysm size within the external dataset, as detailed in Table 3. Consistent with the model's performance when the ratio of aneurysm to normal patch was 1:2, the highest accuracy (0.885) was observed for aneurysm sizes smaller than 5 mm. Especially, our dataset predominantly comprised of very small aneurysms, typically within the 2-3 mm range. The distribution can also be seen in the ROI size histogram shown in Supplement Figure S1. However, we still achieved accuracy of approximately 80% for aneurysms that were smaller than 2 mm.

To overcome the inherent imbalances in the datasets, skeletonization of cerebral blood vessels was used to reduce patch level imbalances between aneurysms and normal regions from the 3D brain-level to 1D skeleton-level. Based on the skeleton, 3D patches were extracted from aneurysms and normal regions. Multi-task learning, which simultaneously executes semantic segmentation and multi-class classification, was proposed to overcome this 1D imbalance between large normal and rare aneurysm regions on the skeletons. While training the U-Net, a semantic segmentation network, with an auxiliary classifier added to the bridge block of U-Net for classification, was used. For training semantic segmentation, the regions with aneurysms, excluding the normal areas, were needed because the segmentation network only learns the aneurysm region. However, there are large regions of normal areas in the skeleton. To overcome this 1D imbalance, we added an auxiliary classifier to differentiate aneurysms from normal patches to improve the differentiation by the deep-learning-based model. Because there is no segmentation region where the auxiliary classifier considers the normal region, a segmentation was performed for those with an annulus region to increase the accuracy. Therefore, the ratio of the normal patches to the aneurysm patches was gradually increased from 1:1 to 5:1. These results ultimately show that segmentation and classification can be taught together in a multi-task learning manner, overcoming the imbalanced datasets. Inserting normal patches without dropping the segmentation accuracy is possible because several normal patches can be shown. Additionally, the external datasets showed no significant difference, with robust model accuracy performance.

**Figure 5:**
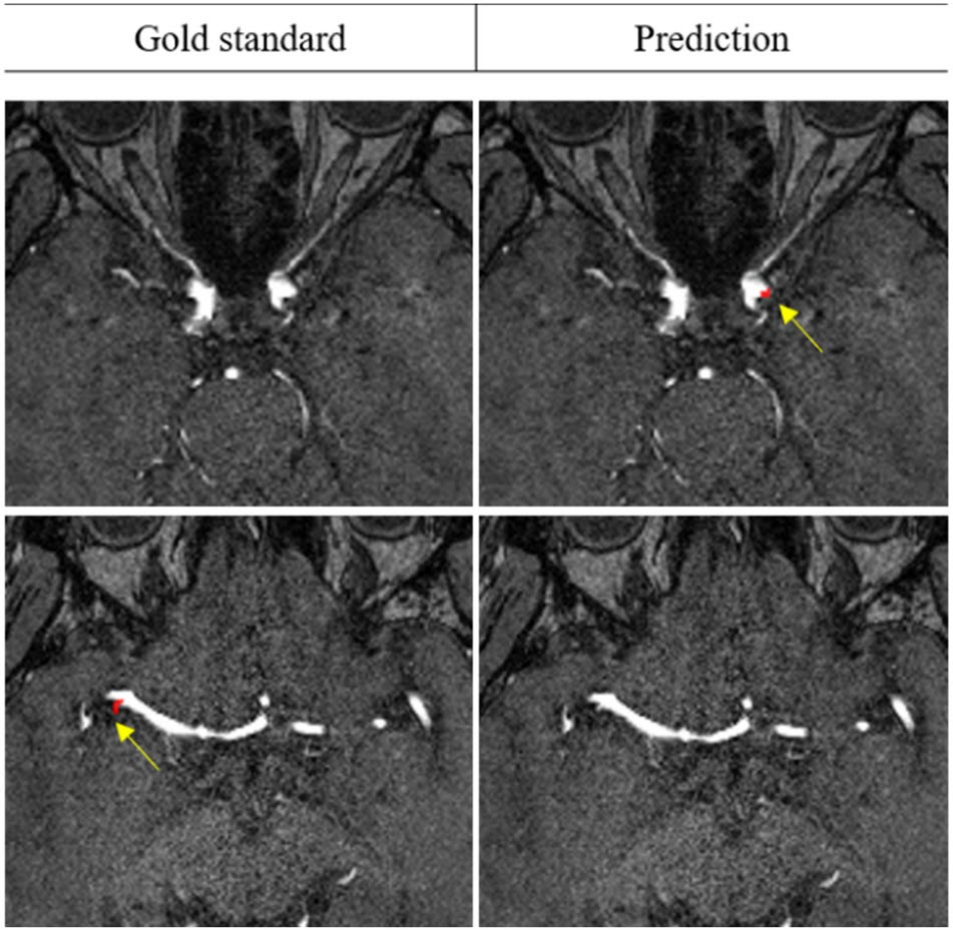
Examples of false positive (top) and false negative (bottom) results.

However, as shown in Fig. 5, there were also cases of poor prediction that resulted in false positives and false negatives on the test set. The causes of the false positives and false negatives appear to be that the size of the aneurysms was much smaller than the trained average size, which may have confused the model and resulted in missed detections, and that the brightness and contrast difference of the aneurysms was not prominent, which caused it to have difficulty detecting the aneurysms.

This study has some theoretical contributions. First, the current study contributes to academia in that it takes a new approach to semantic-segmentation-assisted classification. Previous studies simply performed detection and segmentation on the whole brain image or segmented the aneurysm using pre-processing, for example by using a threshold. In this study, blood vessel segmentation was first performed to detect aneurysms existing along the brain vessels and to access them more easily than by finding 3D points. By combining the labeling of aneurysms along brain vessels with prior anatomical knowledge, we were able to build a powerful deep-learning model. Second, the multi-tasking method enables stable learning for aneurysm images with severe data imbalance. If the data imbalance is severe, most models tend to not learn well, but that did not happen in this experiment. We also strategically analyzed the model's external dataset results by size. This granular analysis allowed us to understand the model's performance in different scenarios, which helped support its generalization performance even when trained with smaller sample sizes.

Our study had several limitations. First, the dataset used in this study is drawn from a single center. In the future, we plan to conduct multi-center studies to test our model across various institutions and devices. This will involve aggregating datasets that were acquired using different imaging protocols and machines, allowing us to investigate the model's performance in a diverse and clinically relevant set of conditions. We anticipate that this will provide a more robust assessment of the model's generalizability and its potential for real-world application. New 3D encoding–decoding network variations should be tested to draw more in-depth implications. Second, the amount of patient data collected for use in the experiment was relatively small. Although severe augmentation was used to increase the training datasets of the aneurysm, more patients should be enrolled for more robust training. Future research should focus on additional training with more patients.

## Conclusion

We generated semantic segmentation models for intracranial aneurysms using 3D patches in brain 3D TOF-MRA via CNN and solved the data imbalance problem. The present study has several implications for the clinical setting. This study supports the possibility of determining the presence of aneurysms along the brain vessels, identifying the boundaries in a short time, and performing segmentation. This 3D patch-level multi-task learning technique, with semantic segmentation and auxiliary classification, showed accurate aneurysm detection in 3D TOF-MRA datasets with good sensitivity and a small FP. Therefore, this model will be helpful for rapid diagnosis in clinical practice.

## Supplementary Information

Supplementary Information.

## Data Availability

The datasets generated and analyzed during the current study are not publicly available for privacy reasons, but are available from the corresponding author on reasonable request.
