# Supplementary material for: Automated detection of intracranial aneurysms using skeleton-based 3D patches, semantic segmentation, and auxiliary classification for overcoming data imbalance in brain TOF-MRA
Source: Sci Rep. 2023 Jul 25;13:12018. doi: 10.1038/s41598-023-38586-9 (PMC10368697; doi:10.1038/s41598-023-38586-9)
Supplement: Supplementary file 1 — Supplementary Information. [file 41598_2023_38586_MOESM1_ESM.docx]

**Supplementary**

Figure S1. Histogram of region of Interest size distribution for internal dataset.

Table S1. The average accuracy for each model depending on patch size.

| Patch size | 3D U-Net | 3D  U-Net with auxiliary  Loss 1:2 | nnU-Net |
| --- | --- | --- | --- |
| 32 | 0.609$\pm$0.088 | 0.693$\pm$0.097 | 0.706$\pm$0.058 |
| 64 | 0.758$\pm$0.068 | 0.859$\pm$0.052 | 0.762$\pm$0.065 |
| 128 | 0.668$\pm$0.064 | 0.735$\pm$0.084 | 0.711$\pm$0.097 |
